# Abdominal Pain as the Manifestation of Chylomicronemia Syndrome During Pregnancy: A Challenging Diagnosis

**DOI:** 10.7759/cureus.14317

**Published:** 2021-04-06

**Authors:** Catarina Parente, Rúben Reis, Manuel Toscano, Isabel Botelho, Armindo Ramos

**Affiliations:** 1 Internal Medicine, Centro Hospitalar Barreiro Montijo, Barreiro, PRT; 2 Internal Medicine, Hospital De Cascais, Cascais, PRT; 3 Internal Medicine: Critical Care, Hospital De Cascais, Cascais, PRT

**Keywords:** chylomicrons, triglycerides, atypical rash, acute pancreatitis, pregnancy, abdominal pain, plasmapheresis

## Abstract

Hypertriglyceridemia is a frequent cause of acute pancreatitis. Levels higher than 1000 mg/dL are often associated with a genetic predisposition that can be aggravated by other factors such as pregnancy and poorly controlled diabetes.

The authors report a 19-year-old primigravida that presented with abdominal pain, emesis and a pruritic rash, along with severely increased plasma triglyceride levels. Therapeutic plasmapheresis was proposed in the setting of a presumed acute pancreatitis.

Chylomicronemia syndrome is a rare and frequently misdiagnosed pathology that can evolve with abdominal pain, vomiting and a specific cutaneous rash designated as eruptive xanthomatosis. This case report illustrates the challenges of achieving a correct diagnosis for rare conditions and corroborates the safety of plasmapheresis during pregnancy.

## Introduction

Abdominal pain is a common symptom frequently approached in the emergency room that can be attributed to multiple causes of distinct clinical severity. One potentially life-threatening condition that manifests with abdominal pain is acute pancreatitis, which is a well-known and frequent entity in clinical practice. Although its diagnosis can be relatively simple, determining the etiology might be challenging. The gold standard image exam for diagnosis of pancreatitis is contrast-enhanced computed tomography (CT), which also allows the exclusion of local complications. In the absence of complicated disease, the initial management usually consists of supportive care with fluid therapy, pain control and restriction of oral intake.

Another frequent cause of abdominal pain that typically occurs with nausea and vomiting is diabetic ketoacidosis, which is generally treated by supportive measures such as glycaemic control, intravenous hydration and correction of electrolytic and acid-base balance disturbances. In pregnancy, a whole other group of diagnostic hypotheses that have to be excluded emerges, including eclampsia and preeclampsia, HELLP (Hemolysis, Elevated Liver enzymes, Low Platelets) syndrome, and uterine contractions amongst others.

A rather rare cause of abdominal pain is chylomicronemia syndrome, which is manifested by a constellation of signs and symptoms: severe elevations in plasma triglycerides, abdominal pain, recurrent episodes of acute pancreatitis, eruptive xanthomatosis and lipemia retinalis [1]. The present case report represents the diagnostic complexity of abdominal pain in the setting of multiple confounding factors and the limitation of certain complementary exams due to gestation or unsuitable blood samples.

## Case presentation

**Figure 1:**
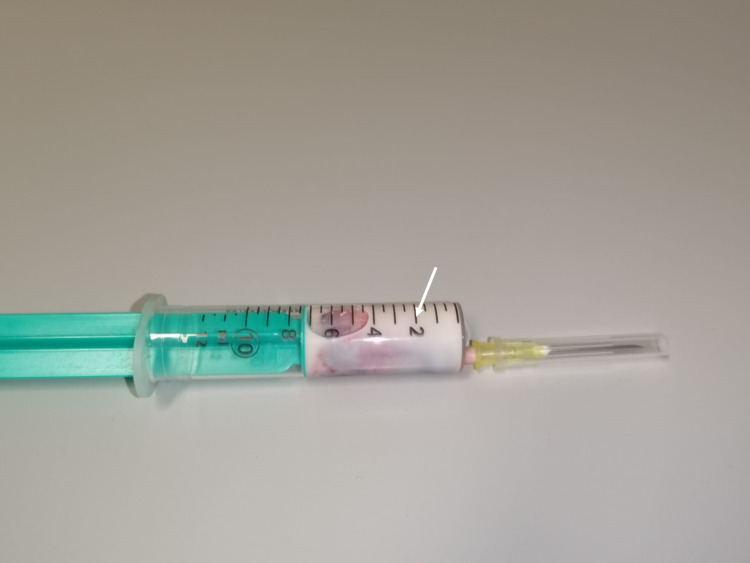
Syringe with blood sample Milky white appearance of collected blood sample (white arrow) from a peripheral vein

A 19-year-old woman with a history of diabetes of unknown etiology, for which there was no therapeutic compliance or follow-up care, presented to the emergency department with nausea, emesis and diffuse abdominal pain. She had an estimated 27-week pregnancy that had not been monitored up to that point. There was no fever or other symptoms. A blood sample was drawn for laboratory analysis which was milky white in the syringe (Figure 1) and results were unobtainable.

Arterial blood gas analysis revealed a pH of 7.12 and bicarbonate level of 3.3 mmol/L, capillary blood glucose was 327 mg/dL and capillary ketone determination was 5 mmol/L. The patient was admitted to the intermediate care unit for diabetic ketoacidosis and started treatment accordingly. Upon admission, a pruritic papular rash was noted in the patient’s legs, feet, arms, hands and trunk.

**Figure 2:**
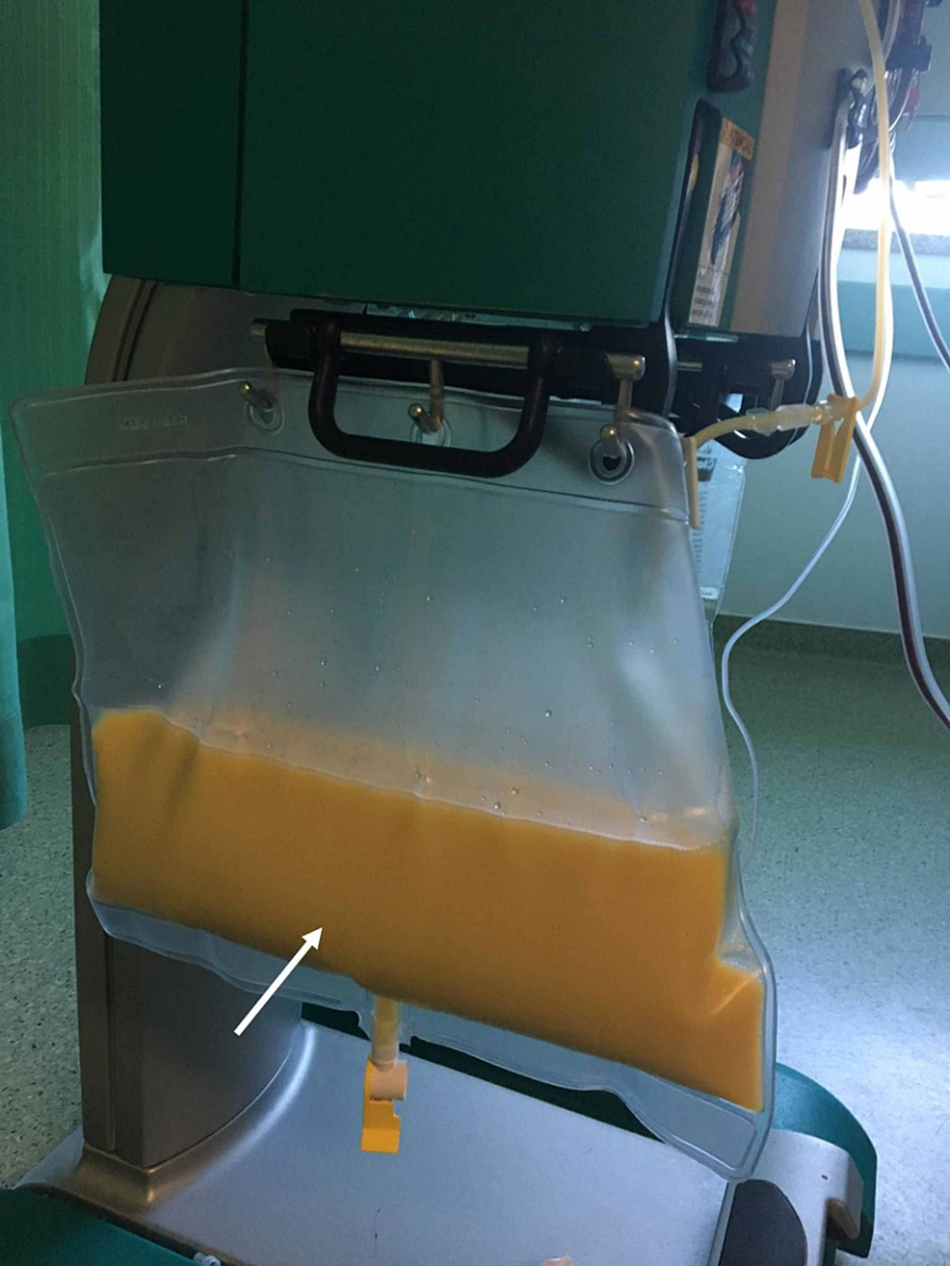
Plasmapheresis system Milky white appearance of plasma (white arrow) in the processing bag

After intravenous fluid therapy, it was possible to perform blood work which demonstrated a glycated haemoglobin level of 12%, plasma amylase of 130 UI/L (reference range: 25 - 115 UI/L) and triglycerides of 9755 mg/dL. Moreover, the patient also had hypercholesterolemia with a total cholesterol of 1065 mg/dL, high-density lipoprotein (HDL) < 20 mg/dL and low-density lipoprotein (LDL) of 374 mg/dL (direct homogeneous enzymatic determination method). There were no detectable changes in abdominal and fetal ultrasound and no contractility in cardiotocography. The case was interpreted as acute pancreatitis due to severe hypertriglyceridemia, and plasmapheresis was conducted (Figure 2). After one session of approximately four hours, her serum triglycerides level was 559 mg/dL and her symptoms resolved. She then started treatment with gemfibrozil.

A hypercholesterolemia study was carried out with apolipoprotein A1 assay value within the reference range and with apolipoproteins E and C-II slightly elevated, 73.6 mg/dL (reference range: 23.0 - 63.0) and 9.5 mg/dL (reference range: 1.6 - 4.2), respectively. It was not possible to measure apolipoprotein B because the blood sample was too lipemic.

The patient was discharged to pursue medical care and investigation in ambulatory regimen but was not compliant to follow-up and further investigation was not possible.

## Discussion

Chylomicronemia syndrome (CS) is a largely misdiagnosed condition characterized by severe elevations in plasma triglycerides, often greater than 1000 mg/dL, and by clinical features as recurrent episodes of abdominal pain that may be associated with nausea and vomiting, most common presenting symptom, acute pancreatitis, lipemia retinalis [1] and/or hepatosplenomegaly. A skin rash may be present in the form of various dispersed papular lesions, yellow, pink or brown coloured, designated as eruptive xanthomatosis which can be pruritic or occasionally painful [2].

There are two forms of CS: familiar chylomicronemia syndrome (FCS), related to mutations affecting the enzyme lipoprotein lipase or its regulators, apolipoprotein C-II, apolipoprotein A5, GPIHBP1 (Glycosylphosphatidylinositol Anchored High Density Lipoprotein Binding Protein 1) and LMF1 (Lipase Maturation Factor 1); and multifactorial chylomicronemia syndrome (MCS) which results from minor polygenic variants and is exacerbated by other medical conditions [3].

Genetic testing for FCS is limited since it is only available at specialized laboratories and it may wrongly dismiss some patients because not all mutations are known [4]. Nevertheless, its diagnosis can be established predominately in the clinical setting [4,5] reuniting the following criteria: severe hypertriglyceridemia (> 880 mg/dL) in three determinations refractory to treatment and diet; history of acute pancreatitis or recurrent abdominal pain, absence of familial history of combined hyperlipidaemia, age of onset of symptoms and exclusion of secondary causes (except pregnancy and ethinylestradiol treatment) [5].

The patient presented with diffuse abdominal pain, nausea and vomiting in the setting of uncontrolled diabetes, namely manifested as diabetic ketoacidosis, and triglycerides level > 9000 mg/dL, associated with a pruritic papular skin rash. Even though eruptive xanthomatosis could not be confirmed due to the absence of a skin biopsy, CS prevailed as the most suitable diagnosis reuniting the presentation features that were, as previously mentioned, severely increased plasma triglycerides, abdominal pain, nausea and vomiting, although some of these symptoms could be attributed to diabetic ketoacidosis. Regardless, acute pancreatitis cannot be excluded since it can occur in the context of CS and considering amylase levels may be falsely normal in the context of hypertriglyceridemia [6]. On the other hand, nonspecific elevations of amylase and lipase can occur in diabetic ketoacidosis [7]. It should be noted that a CT scan was not performed to further investigate this hypothesis due to the potential risks to the foetus. Moreover, all these conditions are not mutually exclusive and can occur simultaneously comprising a rare triad that has been previously described in medical literature.

Despite the well-defined chylomicronemia syndrome (with or without acute pancreatitis), the distinction between FCS and MCS may be more complex. The young age, low body weight and extremely elevated triglyceride levels with a milky white plasma appearance which is a key characteristic of FCS [5] favor this hypothesis. Conflictingly, the patient had uncontrolled diabetes as a precipitating factor and her past medical history was unremarkable for acute pancreatitis. Furthermore, there were no prior triglyceride level determinations.

Considering genetic testing was not immediately available and there was not enough data for using the mentioned clinical criteria, a definitive cause of CS, familiar or multifactorial, could not be concluded.

Another aspect of this case report is the use of plasmapheresis. Therapeutic plasma exchange is a safe and effective technique that can be performed in a broad spectrum of diseases, including during pregnancy, and it is recommended as a second-line therapy in hypertriglyceridemia-induced acute pancreatitis in pregnant women. An important consideration while implementing this procedure in pregnant patients is to ensure a correct estimation of blood and plasma volume (which will be increased especially in the second and third trimesters) [8].

## Conclusions

The reported clinical case was challenging considering that abdominal pain, nausea and vomiting can occur in diabetic ketoacidosis, as well as in acute pancreatitis and in chylomicronemia syndrome manifestation. Amylase levels were borderline normal and a lipase level was not obtained prior to plasmapheresis. Concomitantly, a CT scan was not performed to clarify the diagnosis due to the ongoing pregnancy. Nevertheless, all three conditions could be present simultaneously consisting of a rare but previously described triad in literature. We highlight the diagnostic difficulty of abdominal pain in the presence of confounding factors in the routine clinical practice, as well as the use of plasmapheresis for the treatment of severe hypertriglyceridemia in pregnancy, which was performed without reportable complications.

Furthermore, the authors emphasize that a patient's lack of compliance to treatment and follow-up can be truly limiting for medical action and clinicians should be aware of this problem so that education and sensitization strategies can be developed.
